# The Efficacy of Multiwavelength Red and Near-Infrared Transdermal Photobiomodulation Light Therapy in Enhancing Female Fertility Outcomes and Improving Reproductive Health: A Prospective Case Series with 9-Month Follow-Up

**DOI:** 10.3390/jcm13237101

**Published:** 2024-11-24

**Authors:** Ruth Phypers, Venera Berisha-Muharremi, Reem Hanna

**Affiliations:** 1 Laser Medicine Centre, 134 Harley Street, London W1G 7JY, UK; ruth@lasermedicine.co.uk; 2 Faculty of Medicine, University of Prishtina, Bulevardi i Dëshmorëve nn, 10000 Prishtina, Kosovo; venera.berisha@uni-pr.edu; 3 Poliklinika Endomedica, Muharrem Fejza Str. Nr. 84, 10000 Prishtina, Kosovo; 4 Endocrinology Clinic, University Clinical Center of Kosovo, Lagja e Spitalit, 10000 Prishtina, Kosovo; 5 Department of Restorative Dental Sciences, UCL-Eastman Dental Institute, Medical Faculty, University College London, London WC1E 6DE, UK; 6 Department of Surgical Sciences and Integrated Diagnostics, University of Genoa, 16132 Genoa, Italy

**Keywords:** age-related infertility, ART, fertility, multiwavelength, near-infrared, ovarian rejuvenation, photobiomodulation (PBM), red light, reproductive health

## Abstract

**Background/Objectives**: Female infertility due to unexpected causes exhibits a great challenge for both clinicians and women who are trying to conceive. The present clinical case series study aimed to evaluate the efficacy of multiple wavelengths of red and near-infrared (NIR) laser photobiomodulation (PBM) for increasing the potential of fertility in women and improving reproductive health in unexplained infertility issues. The objectives were to assess the following: (1) any adverse effects; (2) the possibility of producing an effective PBM protocol; (3) and healthy live birth. The inclusion criteria were to related to females who failed to conceive naturally beyond two years, multiple miscarriages, molar pregnancy, non-viable embryos from in vitro fertilisation (IVF) cycles, and failure to complete successful implantation of viable pre-implantation genetic tested (PGT-A) embryos. **Methods**: Case series of three female subjects with unexplained age-related infertility issues, which included a failure to conceive naturally beyond two years, multiple miscarriages, molar pregnancy, non-viable embryos from IVF cycles, and failure to complete successful implantation of viable pre-implantation genetic tested (PGT-A) embryos. In each case, previous conditions were recorded and then compared with outcomes after the patient received a course of PBM treatments. In every case, fertility outcomes improved. Three cases resulted in a full-term pregnancy and the birth of a healthy baby. PBM treatments were given at weekly and/or at two-week intervals using IR and NIR wavelengths between 600 nm and 1000 nm in the lead up to natural conception, IVF oocyte retrieval, blastocyst/embryo implantation, and/or the production of viable embryos. **Results**: In every case, fertility outcomes improved. Improvements in reproductive health outcomes in each case give reason to suggest that PBM may help to improve unexplained age-related infertility. **Conclusions**: Our study demonstrated that multiwavelength of red and NIR PBM with either an LED or laser, or a combination, improved female fertility and reproductive health and contributed to healthy live births in females diagnosed with unexplained age-related infertility. Extensive studies with robust data are warranted to validate our PBM dosimetry and treatment protocols. Moreover, understanding the genetic and phenotype biomarkers is important to standardise a range of PBM light dosimetry.

## 1. Introduction

### 1.1. Infertility Pathogenesis in Females

The pathogenesis of female infertility can be complex. Approximately 25% to 35% of infertile women are found to have tuboperitoneal disease, with pelvic inflammatory disease (PID) being the most common cause of tubal damage [1]. PID is usually a result of Chlamydia trachomatis infections [2]. Bacterial infections can result in tubal occlusion or peritubular adhesions that render in vivo fertilisation unlikely. IVF bypasses the tubal damage by transferring embryos directly into the uterus.

#### 1.1.1. Endometriosis

Endometriosis is confined to the peritoneal or serosal surfaces of the pelvic organs, commonly the ovaries, broad ligaments, posterior cul-de-sac, uterosacral ligaments, and peritoneum. The sites that are less common are as follows: fallopian tubes, rectovaginal septum, and serosal surfaces of the small and large intestines, ureters, bladder, vagina, cervix, and surgical scars [3].

A meta-analysis of 17 studies conducted by Moradi et al., 2021 [4] showed that the prevalence of endometriosis was as follows: 42% in women with chronic pelvic pain; 31% in infertile women; and 18% in reproductive-aged women. It is noteworthy that the timeframe from the onset of the symptoms to the diagnosis of endometriosis ranges between 4 and 11 years old [5], with the average age at the diagnosis being 28 years old [6]. Moreover, angiogenesis is a key mediator of endometrial regeneration, facilitating appropriate nutrient and oxygen delivery, which is important in the treatment of endometriosis and tissue healing [7].

#### 1.1.2. Unexplained Infertility

Unexplained infertility is considered when the semen is normal and the oocytes, ovulation, fallopian tubes, and uterus are also normal. The likelihood of a diagnosis of unexplained infertility is increased substantially in women who are 35 years and older—and greatly increased in women > 38 years because of the increased likelihood of egg quantity and quality issues as women age. Since there is not a “standard category” called egg factor infertility, couples sometimes are lumped into the “unexplained” infertility category. In these cases, there are controversial opinions about the value of endometrial biopsy, ovarian reserve (indicated by AMH and FSH levels), post-coital tests, and serum prolactin, and, in each case, treatments should be individualised [8].

The recommended treatments for unexplained infertility are controlled ovarian stimulation (COS) and in vitro fertilisation (IVF). Due to multifoetal pregnancy associated with COS, the clinician proceeds directly with IVF. The data indicate that women > 38 years with unexplained infertility conceive more quickly and the costs are lower when IVF is performed rather than proceeding to controlled ovarian stimulation [3].

#### 1.1.3. Age-Related Infertility

A woman’s peak reproductive years are between the late teens and late 20s. By the age of 30, fertility starts to decline. This decline increases by the mid-30s, and by 45, fertility has declined to the extent that a natural pregnancy is unlikely [9].

The pathophysiology of age-related fertility decline in females is multifactorial. A decrease in the number of gametes and the integrity of those gametes is affected by age due to oocyte atresia over time, a natural process whereby most ovarian follicles and oocytes die. Furthermore, changes in the meiotic spindle (responsible for the correct segregation of chromosomes) can lead to aneuploidy. Other contributing factors include changes in ovulation patterns with age, the ageing of the uterus, and increasing risks of complications [10].

The mechanisms of decreasing fertility are poorly understood but are most likely to be genetically coded within the X chromosome and the autosomes [11].

Physiological factors that can diminish oocyte quantity and quality include the following: DNA damage, genetic mutations, cohesion deterioration and chromosome missegregation, meiotic recombination errors, spindle assembly checkpoint, telomere shortening, mitochondrial dysfunction, ovarian fibrosis, and inflammation [12].

Although most couples prefer to have a child with genetic material from both parents when possible, oocyte donation may be the best option for some couples. Because aneuploidy rates increase with increasing age, in vitro fertilisation with a woman older than 42 has an 85% risk of aneuploidy [13]. Similarly, a woman aged 35 needs around five oocytes to create a euploid embryo, whereas a woman older than 42 would need around two hundred oocytes. A decreased responsiveness to ovarian stimulation is also seen in older women. When a donor oocyte is used, the success of in vitro fertilisation is based on the donor’s age [13].

Potential complications of age-related fertility decline include a true inability to conceive. The cost of utilising assisted reproductive technology, and the low success rates for older women, are additional complications to keep in mind [9]. Women who give birth aged 34 or older have increased rates of miscarriage, preterm birth, and congenital malformations, especially after the age of 40. Once pregnancy is achieved, complications such as gestational hypertension, preeclampsia, and gestational diabetes are also increased risks during pregnancy [14].

The above factors that contribute to age-related infertility can be related to an overall degenerative ageing of the human body from 35 years of age, which impacts metabolic health and is observed within the female reproductive system. A decrease in mitochondrial energy could impact reproductive health when the health and energy of the individual otherwise appear good [15].

### 1.2. Current Female Fertility Treatments

#### 1.2.1. In Vitro Fertilisation (IVF)

IVF is the second line of treatment for women with unexplained infertility who have not conceived after two years (this can include up to one year before their fertility investigations) of regular unprotected sexual intercourse [16]. It involves the manipulation of oocytes outside the body, termed ART, with IVF as the most common form.

The sequence of the IVF treatment is as follows [17]: (1) controlled ovarian stimulation; (2) oocyte retrieval; (3) embryo fertilisation; (4) and embryo transfer.

IVF is widely regarded as the preferred treatment for unspecified infertility; however, for age-related infertility cases, successful live birth rates are low. According to the 2018 Assisted Reproductive Technology, the live birth rate per intended egg retrieval by age group was as follows [18]: 47.6% for women < 35 years old; 30.7% for women between 35 and 37 years old; 21.7% for women between 38 and 40 years old; 10.4% for women between 41 and 42 years old; and 3.1% for women > 42 years old.

The exploration and development of other fertility treatments, which aim to address unspecified age-related infertility and help improve IVF success rates, have been published. A brief description of these therapies, including their limitations, is given and provides context to our study.

#### 1.2.2. Intra-Uterine/Intra-Ovarian Injection of Platelet-Rich Plasma (PRP)

Intra-uterine autologous platelet-rich plasma (PRP) infusion has been utilised in infertile women with recurrent implantation failure (RIF) and thin endometrial lining thickness (EMT) [19]. A prospective clinical study conducted by Dogra et al., 2022 [20] concluded that PRP enhances EMT significantly during fresh and FET cycles in thin endometrium associated with tuberculosis, polycystic ovary syndrome (PCOS), and diminished ovarian reserve (DOR), thus improving the CPR and LBR in these low prognosis patients. A retrospective observational study conducted by Fraidakis et al., 2021 [21] showed a statistically significant increase in the normal values of follicle-stimulating hormone (FSH) and estradiol (E2) after the PRP intervention. Extensive RCTs are needed to further understand the efficacy of autogenous PRP in ovarian rejuvenation before offering it routinely in clinical practice.

#### 1.2.3. Exosomes Therapy

Exosomes derived from autologous or allogeneic bone marrow-derived stem cells (BMSCs) are utilised to promote uterine repair [22]. The data of an in vitro study conducted by Zhu et al., 2022 [22] provided clear evidence that CTF1 can enhance angiogenesis, suppress tissue fibrosis, and enhance endometrial receptivity following BMSCs-exo treatment, highlighting a viable approach to treating damaged endometrial tissues, but further mechanistic research is required to expand on the pathways whereby C-BMSCs-exosome treatment would be considered effective to achieve therapeutic efficacy.

#### 1.2.4. Ozone Therapy

Ozone has emerged as a new adjunct therapeutic agent for female infertility. The mechanistic effects by which ozone might improve female reproduction are related to its antioxidant and anti-inflammatory properties [23]. The latter collectively reduces the reactive oxygen species (ROS) and interleukin-6 (IL-6) and tumour necrosis factor-α (TNF-α) and increases the following activities [24,25,26]: glutathione peroxidase, superoxide dismutase activity, and antibacterial activity. A recent review conducted by Merhi et al., 2019 [27] and a pilot human study by Merhi et al., 2021 [28] suggested that a combination of ozone sauna therapy (OST) and pulsed electromagnetic field therapy (PEMF) could be a potential therapeutic adjunct for women with endometriosis. The authors concluded that randomised clinical trials are warranted to determine the efficacy of OST and PEMF in women with other inflammatory disorders.

#### 1.2.5. Supplements Treatments

A systematic review of a dietary intake of omega-3 polyunsaturated fats (PUFAs) conducted by Abodi et al., 2022 [29] aimed to summarise the evidence on the effect on oocyte and embryo quality for a positive ART outcome. Another recent systematic review and meta-analysis conducted by Trop-Steinberg et al., 2024 [30] reported that omega-3 intake significantly improves women’s pregnancy and fertilisation rates; however, the high heterogeneity of the studies led to limitations in its interpretation. An increase in oxidative stress (OS) is responsible for premature and decline in ovarian function [31]. In humans, CoQ10 concentrations decrease after 30 years of age in some tissues [32,33] and perhaps contribute to the ageing process.

An in vivo animal study conducted by Ben-Meir et al., 2015 [34] showed that impaired mitochondrial performance created by suboptimal CoQ10 availability can drive age-associated oocyte deficits causing infertility. Hence, CoQ10 can be a promising antioxidant for POR treatment. An RCT conducted by Xe et al., 2020 [35] concluded that (CoQ10) supplementation can increase ovarian response to stimulation and improve oocyte and embryo quality in young, low prognosis patients with diminished ovarian response and be beneficial in clinical pregnancy and live birth.

#### 1.2.6. Acupuncture

Acupuncture can be considered a successful treatment in restoring fertility in patients by improving sperm quality and ovarian function and balancing the endocrine system and hormones [36]. An RCT by Li et al., 2020 [37] was the first large-scale trial that specifically evaluated acupuncture therapy in child-bearing period females with Hashimoto thyroiditis, confirming the effectiveness of acupuncture treatment and showing that it can be employed in clinic setup.

#### 1.2.7. Intravenous Blood Laser Irradiation

The Intravenous Laser Irradiation of Blood (ILIB) was widely used in obstetrics and gynaecology to stimulate utero-placental blood exchange due to its potential effect and positive impact on energy metabolism and cellular health in ageing germ cells [38]. It can modulate oxidative stress (OS) pathways, enhance mitochondrial efficiency, and shift metabolic processes towards more efficient energy production, underscoring its therapeutic promise [39]. A prospective clinical study conducted by Lin et al., 2024 [40] evaluated the effects of ILIB in women aged between 31 and 44 years old with recurrent implantation failure. It concluded that ILIB is a potential approach for enhancing women’s health.

#### 1.2.8. Photobiomodulation Therapy

Our study focuses on the use of photobiomodulation therapy. Photobiomodulation (PBM) is a form of light therapy that encompasses lasers and light-emitted diodes (LEDs) in the visible and non-visible regions of the electromagnetic spectrum within the therapeutic optical window (660–1200 nm) [41]. It embraces the selective activation of anti-inflammatory cytokine pathways and the downregulation of proinflammatory mediators, resulting in a reduction in inflammation [41].

PBM enhances mitochondrial activities, resulting in ATP synthesis, ROS, and Nitric oxide (NO) modulation. Several studies showed that PBM prompts healing and repair [42,43,44], reduces inflammation [41], and alleviates pain [45,46] but its effects on reproductive medicine have not been elucidated [47].

In the context of unspecified age-related infertility, a body of evidence has been explored since the publication of a series of clinical studies in 1996. Several in vitro and in vivo animal studies [47,48,49] have repeatedly shown the effectiveness of PBM for ovarian rejuvenation due to the following benefits to the reproductive system: (1) enhancing mitochondrial activity and ATP production, collectively increasing cellular proliferation and resulting in healthy cells, which are essential for optimal reproductive function; (2) and increasing angiogenesis by enhancing the oxygenated blood flow by upregulating the vascular endothelial growth factor (VEGF), which would enhance the quality of eggs and increase the likelihood of successful fertilisation (OS reduction may enhance the quality of eggs, increasing the likelihood of successful fertilisation); (3) reducing inflammation and oxidative damage by upregulating the anti-inflammatory cytokines and creating a more favourable environment for conception; (4) improving functionality in the scar and surrounding tissues, and creating better movement between layers of the skin, fascia, and abdominal muscles; (5) promoting tissue repair and energetic integrity; (6) and promoting biogenesis and hormonal balance, which are important in fertility issues.

In terms of female fertility, there is evidence to suggest that PBM can improve fertility in women > 35 years old who are affected by lower-quality eggs due to the ageing process [40]. A prospective clinical study was conducted by Grinsted et al., 2022 [50], which reported a 66% pregnancy rate with PBM treatment for 1–3 months in females who struggled with infertility. Another prospective clinical study was conducted by Oshiro et al., 2012 [51], which utilised PBM in Japanese women who were severely infertile and showed that 21.7% of women achieved successful fertilisation after PBM therapy.

With reference to previous PBM clinical studies related to fertility outcomes, this prospective case series study of three cases aimed to evaluate the efficacy of multiple wavelengths of red and near-infrared (NIR) laser PBM for increasing the potential of fertility in women. The objectives were as follows: (1) achieving live birth and (2) establishing a PBM protocol.

## 2. Materials and Method

### 2.1. Study Design

This study involved a prospective observational case series of three patients who presented with primary unexplained age-related infertility. The study was conducted by single experienced laser operator in London, UK between April 2021 and September 2023.

As our study was a case series observational study and not an RCT or a comparative study, we allocated two independent, experienced assessors for data collection and analysis to minimise interobserver variability and bias. All the data were stored in a Microsoft Excel for Mac Version 16.91 Spreadsheet.

The study was conducted in accordance with the Declaration of Helsinki. Informed written consent was obtained from all the patients, and a full explanation of the treatment was provided, including a patient information leaflet. Additionally, informed written consent was obtained from all the subjects regarding publication of their clinical photos, if any, and our study in a scientific peer-reviewed journal.

### 2.2. Eligibility Criteria

#### 2.2.1. Inclusion Criteria

- Females aged between 40–43-year-old with ≥6 months of primary unexplained age-related infertility.
- Subjects with a regular menstrual cycle and a history of frequent miscarriages or failure to conceive.
- Subjects who tried IVF with unsuccessful live birth.
- Subjects who had no systemic diseases.
- Subjects whose partners are healthy, without sperm quality and quantity disorders.
- Subjects with body mass index (BMI) ranged between 20 and 24 kg/m^2^.
- Subjects without sexually transmitted diseases (STDs).

#### 2.2.2. Exclusion Criteria

- Subjects aged >50 years old.
- Subjects with fibroids and/or major lower abdominal surgery, resulting in pelvic adhesions, uterine anomaly, or systemic diseases such as autoimmune disorders.
- Subjects who underwent oncology therapies.
- Subjects who used anticoagulants for plasma infusion.
- Subjects who had received ovarian or uterine PRP therapy.
- Subjects with mental health disorders including active substance abuse or dependence.

### 2.3. Research Focused Question and PICO

The research question was “Does multiwavelength PBM irradiation improve female fertility with live birth?”.

P: Female subjects aged between 40 and 43 years old diagnosed with age-related unexplained infertility [52].
I: PBM of multiple wavelengths: PBM device (660 nm, 810 nm, 850 nm, 880 nm, and 940 nm) and PBM device (660 nm, 800 nm, 905 nm, and 970 nm).
C: Not applicable.
O: Method of pregnancy achieved, ovarian scans, blood tests (fertility tests), blastocyst grading system (IVF).

### 2.4. Interventional Group

Patients’ age (at conception and at birth), weight and height, body mass index (BMI) [53], and skin colour [54] were recorded and stored in Excel sheet by independent healthcare professional who was not involved in the study.

### 2.5. Treatment Protocol and PBM Irradiation Points

The treatment protocol applied PBM at key affected points; it was applied transdermally for penetration of light to 4–5 cm depth to optimise mitochondrial energy.

- Lymph nodes: a low dose short protocol of PBM was applied to four lymph nodes to prepare the system for PBM:
  o Groin: Bilateral inguinal lymph nodes.
  o Clavicle: Bilateral thymus lymph nodes.
- Inverted triangle in the lower abdominal area above the female reproductive system, primarily to target the ovaries, approximately the size of a stretched open hand.
- The area around the naval and upper digestive tract to aid with digestion and waste. PBM improves gut microbiome and digestive function [55], which has a positive effect on genital tract microbiome to aid fertility outcomes [56].
- Lower back, lumbar spine from L3 to the base of sacrum. PBM was applied to target the back of the uterus and lower abdominal area.
- Cervical spine from C1 to T1 to cover the nerves connecting to the thyroid gland and the Vagus nerve and to positively impact the parasympathetic nervous system.

### 2.6. Assessment Tools

1. The method of conception was identified, (a) natural spontaneous conception or (b) IVF, and we recorded the number of eggs retrieved, the number of embryos fertilised, grading of embryos, and PGT-A tests for aneuploidy.
2. Uterine scans

An external uterus ultrasound scan is a non-invasive tool and was employed to assess the progress of pregnancy course by examining foetus’s heartbeat and growth [57].

3. Biochemical Markers

All the subjects had blood tests to evaluate the biochemical markets listed below at eleven weeks of gestation.

- Pregnancy-associated plasma protein A (PAPP-A) is a protein produced by the placenta. It is needed for the implantation process and to maintain a healthy placenta (after birth). It is a genetic biomarker measured as part of the combined pregnancy screening blood test, which is offered around 11–14 weeks of pregnancy. Low levels of PAPP-A [≤0.4 MoM (multiple of the medium)] can be associated with one or more of the following conditions: (1) a baby with lower birth weight; (2) preterm birth; (3) preeclampsia [high blood pressure (hypertension) and protein in the urine (proteinuria)]; (4) and mid-trimester miscarriage [58].
- Embryos with the correct chromosome number are known as euploid. PGT-A is therefore offered to some patients as a treatment to help identify euploid embryos and avoid transferring aneuploid embryos. It was carried out on a Day 5 blastocyst for aneuploidy [59]. For some people, it is advised that a Day 5 blastocyst is frozen and sent for genetic testing to assess its chromosomal accuracy (usually when there have been frequent miscarriages).
- Free beta-human chorionic gonadotropin (hCG) hormone: It is essential to measure the level of the free beta subunit of human hCG hormone in the blood to assess the risk of chromosomal abnormalities and Down’s syndrome in the foetus. Several studies showed that increased levels of second-trimester free β-hCG were associated with a number of pregnancy complications, including late foetal loss, gestational hypertension, pre-eclampsia, intra-uterine growth restriction (IUGR), preterm delivery, and dead foetus in the utero (DFIU). Abnormally low (<0.5 MoM) or high (>2.0 MoM) free β-hCG levels are generally associated with an increased risk of adverse pregnancy outcomes [60].

4. Grading system for blastocyst—Gardner Blastocyst Grading System

**Figure 1:**
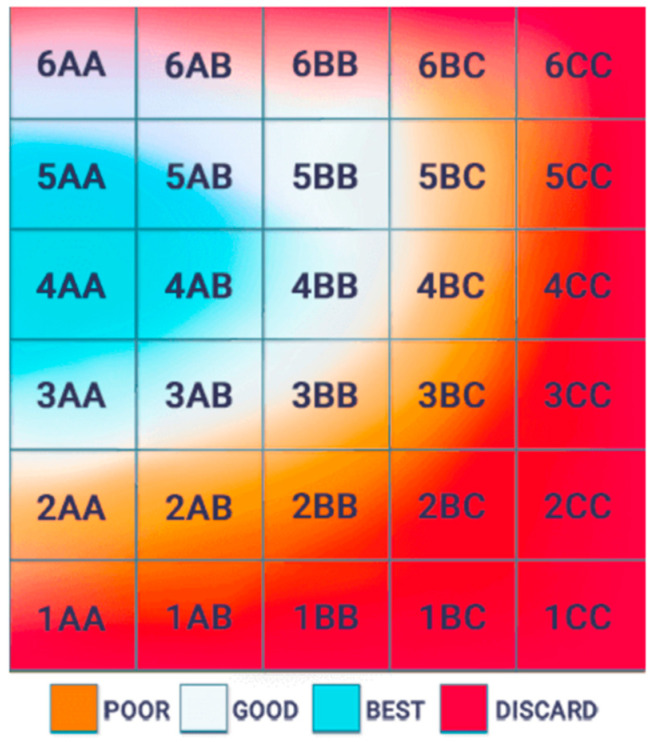
The grading system for blastocyst and success rate, highlighting the hatching days above 3AA as the highest grade, and the hatching days coloured with orange and red are the poor and discard grades [61].

This is the grading system used during IVF to give an indication of the embryo grade with the group of classifications above 3AA being considered good, reflecting the embryo morphology [61]. Figure 1 shows the grades.

### 2.7. Endpoints

#### 2.7.1. Primary Outcome

This study aimed to evaluate the efficacy of PBM (multi-wavelength) therapy to improve female fertility and achieve healthy live births.

#### 2.7.2. Secondary Outcomes

- Reporting any adverse effects.
- Establishing effective PBM and treatment protocols.

## 3. Case Description and Results

### 3.1. Cohort Demographic Characteristics

Table 1 shows the details of the subjects’ age (at conception and at birth), body mass index (BMI), weight, height, and skin colour [54]. All the subjects had a full 9 months of gestation. Two out of three subjects have Type III skin colour based on Fitzpatrick grading. This was considered when the PBM dosimetry and treatment protocol were formulated. Also, subjects’ BMI was taken into consideration. The same dose was delivered with a lower power; therefore, a longer treatment time was employed when the skin was darker.

### 3.2. Cohort Biochemical Markers

All the subjects’ blood results were obtained in their second trimester (at 11 week-gestation) to evaluate the level of PAPP-A and free β-hCG biomarkers. The values of those markers were within the normal range (Table 2), indicating that no health complications were associated with the subjects’ pregnancy.

### 3.3. Case #1

A fit and healthy 41-year-old financially secure woman experienced unexplained age-related infertility issues over the previous 48 months, which included three miscarriages and one molar pregnancy. No contributive medical conditions were presented, apart from a possible COVID-19 infection in January 2020. She was registered on 20 April 2021.

- First Egg Retrieval

A course of six PBM treatments was given in the lead-up to the egg retrieval: 1–2 times per week between 20 April and 24 May 2021, leading to egg retrieval. Table 3 is a representation of the PBM device specifications, dosimetry, and treatment protocols.

The outcome of the retrieval procedure produced four fertilised embryos (22 May 2021). Three embryos progressed to Day 5 blastocysts, of which two blastocysts were genetically tested as normal/euploid (PGT-A).

- Second Egg Retrieval

The time interval between the first and second egg retrieval was two months. A course of twelve PBM treatments with the same dosimetry, as shown in Table 3, was performed between 1 June 2021 and 3 July 2021 before egg retrieval on 23 July 2021.

The outcome of the second retrieval cycle produced six eggs, four fertilised, and one blastocyst went to Day 5, which was genetically tested as normal/euploid.

The first and second egg retrievals resulted in three genetically tested (PGT-A) euploid embryos from two simultaneous egg retrievals.

- Pre-transfer

The final two PBM treatments were given prior to a transfer of one euploid embryo. Table 3 shows the PBM dosimetry, but the number of treatments was two for one week between 25 and 29 October 2021 before embryo transfer. The transfer of one euploid embryo led to a full-term pregnancy and the birth of a healthy baby in July 2022 from the 42-year-old mother.

**Figure 2:**
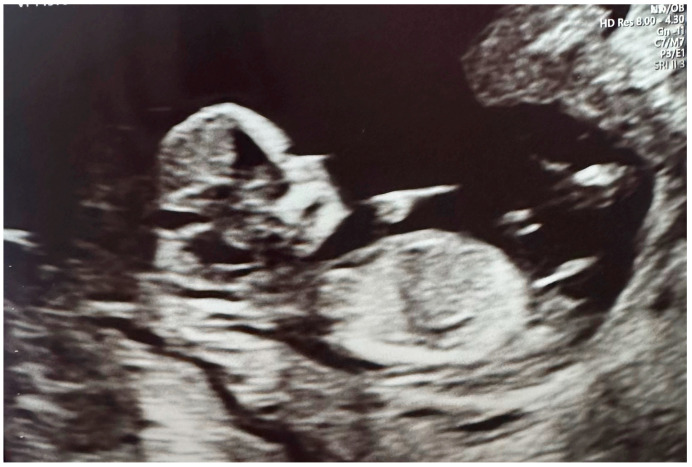
Three-month gestation scan.

Figure 2 shows a 12-week uterine ultrasound scan, confirming key biometry markers indicating a healthy embryo. Foetal biometry: foetal heart rate (FHR) 159 bpm, crown rump length (CRL) 51.7 mm, nuchal translucency (NT) 1.6 mm, biparietal diameter (BPD) 21.3 mm, brainstem (BS) 2.6 mm, brainstem occipital bone (BSOB) 4.4 mm, BS/BSOB 0.591. Foetal anatomy: skull normal, spine normal limited, heart normal limited, stomach visible left-sided, abdomen normal, bladder visible, upper extremities both seen, lower extremities both seen. Additional markers for aneuploidies: nasal bone present, normal tricuspid flow, Ductus Venosus PIV: 0.790.

### 3.4. Case #2

A 40-year-old fit and healthy female experienced unexplained age-related infertility issues after trying to conceive for two years. She underwent four IVF cycles during this time, which produced two blastocysts and one miscarriage at week 12.

A course of PBM treatments was given during the stimulation period of a fifth IVF cycle (from Day 1–22, November 2021) at 2–3 daily intervals (Table 4). Five treatments of PBM between 22 November 2022 and 5 December 2022 were performed leading up to egg retrieval.

A fresh transfer of the Day 3 blastocyst resulted in a successful implantation and a full-term pregnancy, leading to the birth of a healthy baby boy in August 2023 from the 41-year-old mother.

First-trimester screening report on 10 February 2023 confirmed embryo-foetal biometry: FHR 158 bpm, CRL 52.4 mm, NT 1.40 mm; foetal anatomy: skull normal, spine normal, heart normal limited, stomach visible left-sided, abdomen normal, bladder visible, upper extremities normal, lower extremities normal; additional markers for aneuploidies: nasal bone present, normal tricuspid flow, Ductus Venosus PIV: 1.080.

### 3.5. Case #3

A fit and healthy 42-year-old financially secure woman failed to conceive or sustain a successful pregnancy and experienced two miscarriages over a 3-year period. There were no medical conditions presented, and unexplained age-related infertility was assigned. The patient underwent one IVF cycle in the UK and four IVF cycles outside of the UK, which resulted in one genetically tested (PGT-A) euploid kept in storage.

- Egg Retrieval

A course of twelve PBM treatments was given in a period of 2–3 months during the lead-up to the next planned IVF cycle at mostly one-week intervals between 13 December 2022 and 22 March 2023. The outcome of the retrieval procedure collected eight eggs: five mature and three medium-quality eggs. Seven eggs were fertilised to develop into six high-grade Day 5 blastocysts (more than twice retrieved and fertilised during the previous IVF cycle two years earlier). Table 5 illustrates the PBM dosimetry and treatment protocols.

After preimplantation genetic testing (PGT-A), all blastocysts were indicated as aneuploid.

- Pre-transfer and PBM Protocol

Six PBM treatments (Table 6) were given prior to a transfer of one stored euploid embryo between 19 April 2023 and 29 May 2023 before embryo transfer, which did not result in a pregnancy.

- Planned Natural Conception

A further three PBM treatments (Table 7) were given to support a natural conception, between 5 July 2023 and 26 July 2023, which immediately led to a natural conception and a positive pregnancy test.

- First Trimester PBM Treatments

In this case, we agreed to give PBM in order to support the patient’s mitochondrial energy during the first trimester. The patient had a long history of infertility, with repeatedly poor IVF outcomes. We provided PBM to support the first trimester of pregnancy by improving mitochondrial energy to optimise early embryonic development.

This was a different protocol (Table 8), avoiding the abdominal area completely, and instead applying PBM to the following areas: cervical spine, bilateral thymus lymph nodes, axillary lymph nodes, and sole of the feet. The six PBM LED treatments were given between 16 August 2023 and 27 September 2023, taking the pregnancy to 12 weeks.

**Figure 3:**
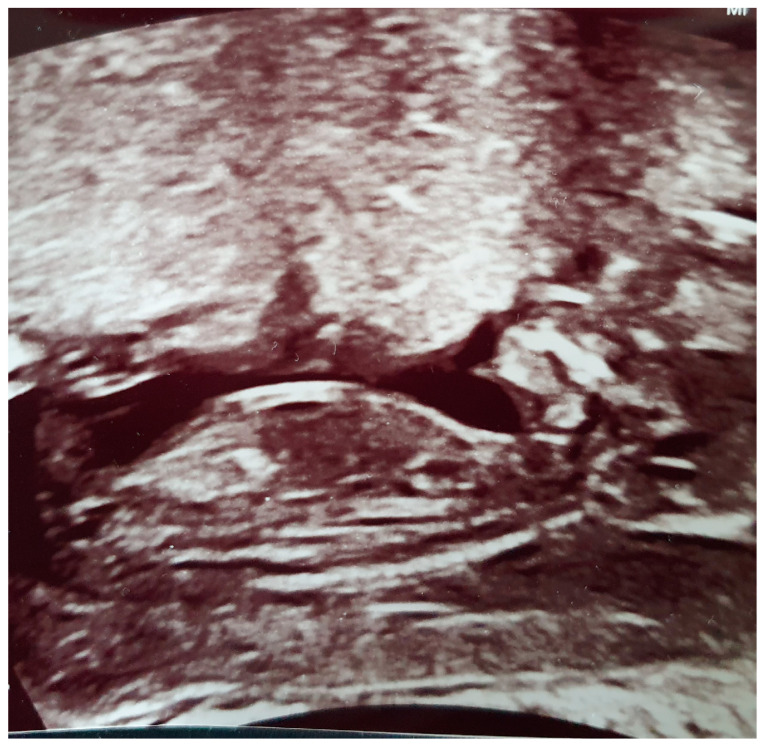
Twelve-week gestation scan.

It is noteworthy that a systematic review conducted by Wilkerson et al., 2019 [62] highlighted that cutaneous laser therapy treatments are safe for both the mother and the foetus. Given the thickness of the pregnant abdomen (30 mm on average), the uterus, and amniotic fluid, clinically meaningful laser energy is very unlikely to reach the foetus. Hence, Figure 3 shows a 12-week scan confirming a healthy embryo with foetal biometry and foetal anatomy markers within range, and the additional markers for aneuploidies were also normal.

The pregnancy progressed to full-term with no complications and resulted in the birth of a healthy baby boy in April 2024 from the 43-year-old mother.

Table 9 illustrates the summary of the utilised PBM light devices specifications, PBM dosimetry and treatment protocols, and patients’ outcomes.

## 4. Discussion

Ageing is associated with a decrease in mitochondrial function, especially in nonreplicating cells such as mature oocytes, and it is considered the basis of declining rates of fertility in women [63,64,65]. Hence, PBM is considered an effective therapy for unexplained age-related infertility due to an up-stream and down-stream biological chain of events that regenerate tissue, improve angiogenesis, and increase mitochondrial energy by increasing ATP synthesis in the eggs [63]. This was well demonstrated in our study when multiwavelength of red and NIR PBM light were utilised, resulting in enhanced fertility and healthy live births in all four subjects [66].

The link between the scientific literature and the interpretation of our findings, including future research directions, is outlined below.

### 4.1. Mitochondrial Bioenergetic and PBM in Fertility

Mitochondrial (mt) efficiency is related to an accumulation of mutations in mtDNA, which may limit energy production. As a result, the cell has a decreased capacity to support all cellular events, especially normal chromosomal segregation during cell division. Also, the implantation process needs a lot of energy and would therefore be compromised by reduced ATP production. In this context, in stressed cells, the NO produced in the mitochondria is connected to cytochrome c oxidase; therefore, it competitively moves O_2_, inhibits electron transport, and disrupts the respiratory chain [67]. On this note, Hamblin et al., 2017 observed that ROS levels decreased under cellular OS situations in animal disease models after PBM [41].

All of the above factors explain that the role of mitochondrial activation in oocyte biology is related to ATP levels peaking during polar body extrusion and being effective in meiosis. In humans, higher ATP levels in oocytes correlate with better embryo development and implantation rates [68]. This was reinforced by a study conducted by Babayev et al., 2015 [15], which showed that the human oocyte contains more mitochondria than any other cell in the body. So, in cases of meiotic division errors, mitochondrial DNA mutations and ageing itself have also been suggested to play a part in the age-associated reduction in oocyte quality [69]. As women become older, they seem to have more mitochondrial DNA mutations which can be responsible for poor implantation and aneuploidy [70]. Hence, it is logical to think that harnessing PBM therapy to increase ATP synthesis and production in the oocyte mitochondria can improve oocyte function and support the production of a viable embryo, and this was well demonstrated in the present study where all the subjects had a healthy pregnancy and live birth. With this in mind, the present study’s PBM protocol was effective in improving mitochondrial energy in the ovum and the subsequent creation of a blastocyst/embryo and enhancing the capacity of the egg to fulfil its function [60]. Hence, mitochondrial activation is one of the reported beneficial effects of PBM, including systemic blood flow increase. Therefore, PBM-mediated mitochondrial activation might contribute to the favourable response to ART and increase the probability of pregnancy.

### 4.2. Fertility and PBM Dose–Response

The optical properties of the irradiated target tissue are of importance when PBM dosimetry is chosen [71]; the wavelength that determines the depth of the tissue penetration is also important [72]. When looking at female fertility specifically, the primary targets are the uterus, ovaries, fallopian tubes, general hormonal systems (thyroid, brain, etc.) [73], and lymphatic chains; hence, the choice of light type with the optimal penetration is necessary.

Several studies showed the effectiveness of NIR on female fertility [50,51,74]. Moreover, red PBM light can offer higher implantation rates, live birth rates, and lower miscarriage rates by increasing the expression of genes tied to embryo implantation. This is materialised through a mechanism by which PBM enriches and improves the blood supply to the target tissue, resulting in VEGF upregulation and an improvement in oxygen and nutrient deliveries.

Of the cells in the body, egg cells showed to benefit from red and NIR light therapy more than any other cells due to the processes of mitosis and meiosis, which need a volume of ATP that is essential for reproductive health [74,75]. Hence, in our study, we employed multiwavelength red and NIR PBM light therapy, as it offers various wavelengths corresponding to various photoacceptors, and each wavelength penetrates the skin at a different depth. Also, in our treatment protocols, we considered the biological activity exerted on the irradiated cells. This is particularly relevant to patients with unexplained infertility.

Our PBM dosimetry and treatment protocols ensured optimal dose delivery to the deep-seated target tissues to modulate the cellular and molecular activities in restoring the mitochondrial DNA of the eggs through the activation of mitochondrial function. In case #1, the LED dosimetry and treatment protocol utilised (Table 3) were sufficient for the first egg retrieval, which resulted in the production of four fertilised embryos, of which three progressed to Day 5 blastocysts, of which two were genetically tested as normal/euploid (PGT-A). The same protocol was employed in case #3 in the first trimester to boost the patient’s immune response, particularly through the role of pro- versus anti-inflammatory cytokines [76]; stimulate cellular function; promote circulation in reproductive organs; and alleviate stress during uncertain times.

The PBM dosimetry for LED and laser PBM and treatment protocols in the present study were in agreement with the study conducted by Grinsted et al., 2019 [50], which evaluated the efficacy of PBM in improving fertility. Approximately 400 women aged between 34 and 50 years old with a diagnosis of infertility (who had tried dietary changes, exercise, hormonal treatments, IUI, IVF, and ICSI) had been treated with PBM, of whom 260 (≈65%) became pregnant and had healthy live births. The PBM dosimetry and treatment protocols were as follows: at the first day of menstruation; a total of 6 PBM treatments over the course of 2 weeks with attempted insemination occurring at day ~12–14 (with ovulation); if conception did not occur (natural or artificial), another treatment course started on the first day of the next menstruation; 23 min of 20,000 J (15k joules of near-infrared light at 808 nm plus 5k J of red light at 660 nm) placed 1–2 cm above the skin with an area of 500 cm^2^. They concluded that “PBM could be a viable choice as a natural, non-invasive addition to other methods of ART”.

The application of PBM to the entire female reproductive system, including the fallopian tubes, and uterus, could improve tissue integrity and oxygenated blood flow and further contribute to the possibility of a full-term pregnancy and healthy birth [50,51]. Moreover, the irradiation points that were employed to boost patients’ immune systems, enhance circulation, and improve mood, resulting in a better chance of conception, are as follows: lower back; sacrum; cervical spine; bilateral axillary lymph nodes; bilateral thymus lymph nodes; sole of the feet; above ovaries/uterus/abdomen. These points are well explained by a Japanese study [51] based on the “proximal priority technique”, taking into account that the brain (specifically the hypothalamus and anterior/posterior pituitary) is the “master hormonal regulator” of the body. This technique targets the central application of PBM to have a downstream release of hormones and effects. Ohshiro, in 2012 [51], developed the “Proximal Priority Theory” using laser NIR (830 nm) PBM on the neck of infertile women with a history of severe infertility and unsuccessful ART (over 9.13 years on average) between 1996 and 2012 (total of 701 women). The total number of treatment sessions was on average ~21. Upon the completion of PBM, pregnancy was achieved in 156/701 patients (22.3%), resulting in 79 live births (79/156 clinical pregnancies, or 50.1%). No adverse effects were noted. They concluded that the irradiation of the neck/upper vasculature with 830 nm at 60 mW led to an improvement in women’s fertility and healthy live births by improving angiogenesis (increasing lymphatic flow), regulating inflammation, decreasing stress, accelerating tissue repair and healing, improving the gut microbiome, and improving the brain’s altered states such as mood, focus, and libido. Our findings are in agreement with Ohshiro et al., 2012 [51].

Moreover, another study conducted by El Faham et al., 2018 [66] postulated that applying PBM to the lower abdomen can improve the proliferative and functional capacity of endometrial cells of the inner lining of the uterus (which can be helpful for embryo transfer success). Our findings are in agreement with El Faham et al., 2018 [66].

The treatment protocols used in cases #1 and #2 supported a straightforward adjunct to IVF egg retrieval and the formation of embryos, which were transferred as a tested PGT-A euploid embryo (FET) in case #1 and the fresh transfer of a Day 3 embryo in case #2. The difference in the dosimetry of case #1 and case #2 could be addressed by considering the protocol length and frequency of PBM sessions. The PBM protocol for case #1 was to give a lower dosimetry of light for a longer period of time before egg retrieval. Weekly/biweekly sessions were given for one month preceding the first IVF cycle, and a further two months before the second IVF cycle. Case #2 received a considerably higher dosimetry for fewer sessions during the stimulation days leading up to an egg retrieval. It is of note that case #2 experienced several failed IVF cycles before the successful retrieval and transfer of a fresh Day 3 embryo, and case #1 experienced repeated miscarriages and a molar pregnancy, which are, in both cases, indications of age-related infertility.

The protocol leading up to the spontaneous conception and live birth for case #3 was lengthy, spanning several months from the first treatment to the last. With no underlying health issues for an extremely fit and healthy 42-year-old woman, the underlying cause of infertility was probably age-related. The results of the egg retrieval carried out after 17 PBM sessions in total, all delivered at the dosimetry protocol of 12,600 J per session, supported the retrieval of seven fertilised embryos, six of which developed to Day 5. The patient reported that this was the most successful IVF cycle they had experienced, yet all embryos were still tested as PGT-A aneuploid. More PBM sessions were given in the lead-up to what resulted in the failed transfer of one euploid embryo, which could arguably have supported the eventual spontaneous conception and healthy live birth.

It is important to note that although the creation of effective PBM dosimetry and protocols can assume certain standards, each protocol is unique to the individual’s needs.

### 4.3. Role of PBM and ART (IVF) for Healthy Conception

Severe female infertility is almost always related to the malfunction of the ovaries. ART is important for overcoming infertility and IVF has been known for decades as the last resort for conception when natural conception fails. However, the cost of IVF per cycle can be very high, even unfeasible for many couples, and the success rates can be very low, especially in women aged ≥35 years old. Nevertheless, a combination of PBM and ART can improve fertility in the latter age group of women [51].

A clinical study by Ohmaru et al., 2012 [77] investigated the effects of PBM-assisted ART on a total of 337 females with severe infertility. Of these, 110 cases became pregnant and the fertility rate was 32.6% for patients receiving ART and PBM. They suggested that PBM with conventional assisted treatments can lead to an increased fertility rate for ART. Our findings are in agreement with the latter, but our PBM protocol showed positive pregnancy rates and healthy live births in all the subjects. This could be related to our small sample size and hence further studies are warranted to validate this.

A study conducted by Huang et al., 2011 [78] investigated whether ART alone is more effective compared to a combination of PBM and ART in enhancing fertility and increasing pregnancy rates. Their results showed that, in relation to the age category, the pregnancy rate was expressed as a lower percentage when undergoing ART alone and increased in those for whom ART and PBM therapy were combined. Hence, it showed that ART alone for women in their later 20s is very effective, but in all other age groups that were <45 years old, a combination of PBM and ART is the better approach. Our study findings are in agreement with the latter.

PBM with red and NIR light operates directly on the mitochondria, improving function and reducing issues like DNA mutations. This explains why a study conducted by Grinsted et al., 2019 [50] showed that two-thirds of women who previously had failed IVF cycles achieved a successful pregnancy (even natural pregnancies) with light therapy. Our findings are in agreement with Grinsted et al., 2019 [50].

In the early-stage embryo, the mitochondria existing within the oocyte must provide adequate ATP to fuel the first few days of embryonic development [64,65]. This was supported by many authors who postulated that the mitochondria have a determinant role in oocyte maturity and the subsequent fertilisation rate [79,80,81]. The oxidative phosphorylation process is crucial for embryo development and plays a role in blastocyst differentiation, expansion, and hatching [82]; hence, PBM plays a fundamental role in establishing a balance between mitochondrial dysfunction, oocyte and biogenesis, and homeostasis of female reproductive health [46,47,51]. The findings of the present study demonstrated the effects of PBM in enhancing fertility and the rate of healthy live births.

### 4.4. Study Limitations and Future Scope

Despite the fact that our study is a case series, the significant results demonstrated the efficacy of multiwavelength PBM with LEDs or laser, or a combination, in improving the entire reproductive system and enhancing oxygenated blood flow, which resulted in full-term pregnancies and healthy live births. Nevertheless, it is important to highlight our study’s limitations, which are as follows: (1) a lack of a controlled longitudinal observational study on a series of subjects receiving the same intervention (i.e., there was no control/sham group); (2) a low number of treated subjects; (3) level IV evidence-based medicine; (4) little statistical validity due to a lack of the control group to compare the outcomes; and (5) a lack of a quantitative assessment tool.

## 5. Conclusions

To the authors’ best knowledge, this is the first study demonstrating that multiwavelengths of red and NIR PBM LED or laser, or a combination, could improve female fertility and reproductive health, resulting in a healthy live birth in females diagnosed with unexplained infertility. Extensive studies with robust data are warranted to validate our PBM dosimetry and treatment protocols. Moreover, understanding the genetic and phenotype biomarkers is important to standardise a range of PBM light dosimetry.

## Figures and Tables

**Table 1:** Presentation of subjects’ demographic characteristics.

Case #	Age at Conception (yrs)	Age at Birth (yrs)	Weight (Pre-Conception) (Kg)	Height (cm)	BMI	Skin Colour
1	41	42	60	160	23.4	III
2	40	41	61	170	21.1	II
3	42	43	66	177	21.1	III

**Table 2:** The maternal serum biochemistry markers associated with foetal growth at 11 weeks of gestation. Kryptor compact (Brahms) was employed for the analysis. Abbreviations: MoM: multiple of the medium; Free β-hCG: free Beta-human chorionic gonadotropin.

Case #	Free β-hCG (MoM)	PAPP-A (MoM)
Case #1	0.6012	0.5031
Case #2	0.6125	0.5889
Case #3	0.6513	0.512

**Table 3:** Device specifications, LED PBM dosimetry, and treatment protocols. Abbreviations: LED: light-emitted diode; W: watt; J: joule; mins: minutes; Hz: hertz; nm: nanometre.

Manufacturer	Omega XP
Semiconductor materials (emitter type)	GaAIAs
Probe design	60 diodes multiwavelength cluster LEDs 24 × 660 nm 10 mW LEDs 12 × 810 nm 20 mW LEDs 12 × 940 nm 20 mW LEDs 6 × 880 nm 20 mW LEDs 6 × 850 nm 20 mW LEDs
Device classification	3b laser or LED
Beam profile	Gaussian
Laser-aiming beam	None
Wavelength (nm)	660, 810, 850, 880, 940
Operating emission mode	Pulsed wave (variable between 146 Hz and 5 kHz)
Polarisation	Linear
Therapeutic power output (mW)	960
Fluence (dose) (J/cm^2^)/point	30
Irradiation time per point (mins)	5
Total number of irradiated points above ovaries and uterus	6
Total dose per session (J/cm^2^)	180
Total irradiation time/session (min)	30
Time interval	Approximately 4 days
Treatment frequency	Once or twice per week
Total treatment sessions	6
Treatment duration	5 consecutive weeks
Scanning technique	Stationery application
Light skin tissue distance	In contact

**Table 4:** Device specifications, laser PBM dosimetry, and treatment protocols. Abbreviations: CW: continuous wave; SP: super pulsed; s: second.

Manufacturer	K-Laser
Semiconductor materials (emitter type)	GaAIAs
Probe design	Probe with 4 wavelengths
Device classification	Type 4 laser
Beam delivery system	Fibre
Beam profile	Gaussian
Laser-aiming beam	None
Wavelength (nm)	660, 800, 905, 970
Operating emission mode	Combined CW and SP
Polarisation	Linear
Therapeutic power output (W)	15
Fluence (dose) (J/cm^2^)	3150
Irradiation time per dose	5 min and 15 s
Total number of irradiated points per session to ovaries/uterus/abdomen	3
Total number of irradiated points per session to lower back/sacrum/cervical spine	1
Total of fluence (J/cm^2^) per session	12,600
Total irradiation time per session	20 min and 45 s
Time interval	Approximately 3 days
Treatment frequency	Twice per week
Total treatment sessions	5 sessions
Treatment duration	2 weeks
Scanning technique	In motion application
Light skin tissue distance (cm)	4.5

**Table 5:** Device specifications, laser PBM dosimetry, and treatment protocols.

Manufacturer	K-Laser
Semiconductor materials (emitter type)	GaAIAs
Probe design	Probe with 4 wavelengths
Device classification	Type 4 laser
Beam delivery system	Fibre
Beam profile	Gaussian
Laser-aiming beam	None
Wavelength (nm)	660, 800, 905, 970
Operating emission mode	Combined CW and SP
Polarisation	Linear
Therapeutic power output (W)	15
Fluence (dose) (J)	3150
Irradiation time per dose	5 min and 15 s
Total number of irradiated doses above ovaries/uterus/abdomen	3
Total number of irradiated doses through lower back/sacrum cervical spine	1
Total of fluence per session (J/cm^2^)	12,600
Total irradiation time per session	20 min 45 s
Time interval	Approximately 10 days
Treatment frequency/duration	Once per week/two weeks
Total treatment sessions	Twelve sessions
Treatment duration	3 months
Scanning technique	In motion application
Light skin tissue distance (cm)	4.5

**Table 6:** Device specifications, laser PBM dosimetry, and treatment protocol.

Manufacturer	K-Laser
Semiconductor materials (emitter type)	GaAIAs
Probe design	Probe with 4 wavelengths
Device classification	Type 4 laser
Beam delivery system	Fibre
Beam profile	Gaussian
Laser-aiming beam	None
Wavelength (nm)	660, 800, 905, 970
Operating emission mode	Combined CW and SP
Polarisation	Linear
Therapeutic power output	15 W
Fluence (dose) (J/cm^2^)	3150
Irradiation time per dose	5 min 15 s
Total number of irradiated doses above ovaries/uterus	3
Total number of irradiated doses through lower back/sacrum/cervical spine	1
Total of fluence per session (J/cm^2^)	12,600
Total irradiation time per session	20 min and 45 s
Time interval	Approximately 3 days
Treatment frequency	Once per week
Total treatment sessions	6
Treatment duration (week)	6
Scanning technique	In motion application
Light-tissue distance (cm)	4.5

**Table 7:** Device specifications, laser PBM dosimetry, and treatment protocol.

Manufacturer	K-Laser
Semiconductor materials (emitter type)	GaAIAs
Probe design	Probe with 4 wavelengths
Beam delivery system	Fibre
Device classification	Type 4 laser
Beam profile	Gaussian
Laser-aiming beam	None
Wavelength (nm)	660, 800, 905, 970
Operating emission mode	Combined CW and SP
Polarisation	Linear
Therapeutic power output (W)	15
Fluence (dose) (J/cm^2^)	3150
Irradiation time per dose	5 min and 15 s
Total number of irradiated doses above ovaries/uterus/abdomen	3
Total number of irradiated doses through lower back/ sacrum/cervical spine	1
Total of fluence per session (J/cm^2^)	12,600
Total irradiation time per session	20 min and 45 s
Time interval (day)	7
Treatment frequency	Once per week
Total treatment sessions	3
Treatment duration (week)	3
Scanning technique	Moveable application
Light skin tissue distance (cm)	4.5

**Table 8:** Device specifications, LED PBM dosimetry, and treatment protocol.

Manufacturer	Omega XP
Semiconductor materials (emitter type)	GaAIAs
Probe design	60 diodes multiwavelength cluster
Device classification	Type 3b laser or LED
Beam delivery system	Fibre
Beam profile	Gaussian
Laser-aiming beam	None
Wavelength (nm)	660, 810, 850, 880, 940
Operating emission mode	Pulsed wave (varied between 146 Hz and 5 kHz)
Polarisation	Linear
Therapeutic power output (mW)	960
Fluence (dose) (J/cm^2^)	6
Irradiation time per point (mins)	1
Total number of irradiated points cervical spine (4), bilateral axillary lymph nodes (2), bilateral thymus lymph nodes (2), sole of the feet (2)	10
Total of fluence per session (J/cm^2^)	60
Total irradiation time per session (mins)	10
Time interval	7 days
Treatment frequency	Once per week
Total treatment sessions	6
Treatment duration	6 weeks
Scanning technique	Stationery application
Light skin tissue distance	In contact

**Table 9:** Summary table of device specification, laser PBM dosimetry, treatment protocols, and patient outcomes.

PBM Light Device Specifications, PBM Dosimetry, PBM Treatment Protocols, and Outcomes		Case 1			Case 2	Case 3			
		First Egg Retrieval	Second Egg Retrieval	Pre-Transfer	Egg Retrieval	Egg Retrieval	Pre-Transfer	Planned Natural Conception	First Trimester
**Dosimetry**	Manufacturer	Omega XP			K-Laser	K-Laser	K-Laser	K-Laser	Omega XP
	Semiconductor	GaAIAs			GaAIAs	GaAIAs	GaAIAs	GaAIAs	GaAIAs
	Probe design	60 diodes multiwavelength cluster LEDs			Probe with 4 wavelengths	Probe with 4 wavelengths	Probe with 4 wavelengths	Probe with 4 wavelengths	60 diodes multiwavelength cluster LEDs
		24 × 660 nm 10 mW LEDs							24 × 660 nm 10 mW LEDs
		12 × 810 nm 20 mW LEDs							12 × 810 nm 20 mW LEDs
		12 × 940 nm 20 mW LEDs							12 × 940 nm 20 mW LEDs
		6 × 880 nm 20 mW LEDs							6 × 880 nm 20 mW LEDs
		6 × 850 nm 20 mW LEDs							6 × 850 nm 20 mW LEDs
	Device classification	3b laser or LED			Type 4 laser	Type 4 laser	Type 4 laser	Type 4 laser	3b laser or LED
	Beam Profile	Gaussian			Gaussian	Gaussian	Gaussian	Gaussian	Gaussian
	Beam Delivery System	N/A			Fibre	Fibre	Fibre	Fibre	N/A
	Laser-aiming beam	None			None	None	None	None	None
	Wavelength (nm)	660, 810, 850, 880, 940			660, 800, 905, 970	660, 800, 905, 970	660, 800, 905, 970	660, 800, 905, 970	660, 810, 850, 880, 940
	Operating emission mode	Pulsed wave (variable between 146 Hz and 5 kHz)			Combined CW and SP	Combined CW and SP	Combined CW and SP	Combined CW and SP	Pulsed wave (variable between 146 Hz and 5 kHz)
	Polarisation	Linear			Linear	Linear	Linear	Linear	Linear
	Therapeutic power output (mW)	960			15	15	15	15	960
	Fluence (dose) (J/cm^2^)/point	30			3150	3150	3150	3150	6
	Irradiation time per point (mins)	5 min			5 min 15 s	5 min 15 s	5 min 15 s	5 min 15 s	1 min
	Total number of irradiated points above ovaries and uterus	6			3	3	3	3	N/A
	Total number of irradiated points to lower back/sacrum/cervical spine	N/A			1	1	1	1	N/A
	Total number of irradiated points to cervicalspine (4), bilateral axillary lymph nodes (2), bilateral thymus lymph nodes (2), sole of the feet (2)	N/A			N/A	N/A	N/A	N/A	10
	Total dose per session (J/cm^2^)	180			12,600	12,600	12,600	12,600	60
	Total irradiation time/session	30 min			20 mins45 s	20 min and 45 s	20 min and 45 s	20 min and 45 s	10 min
	Time interval	Approximately 4 days	Approximately 3 days	Approximately 3 days	Approximately 3 days	Approximately 10 days	Approximately 3 days	7 days	7 days
	Treatment frequency	Once or twice per week	Twice per week	Twice per week	Twice per week	Once per week two weeks	Once per week	Once per week	Once per week
	Total treatment sessions	6	12	2	5	12	6	3	6
	Treatment duration	5 consecutive weeks	5 consecutive weeks	1 week	2 weeks	3 months	6 weeks	3 weeks	6 consecutive weeks
	Scanning technique	Stationery			In motion	In motion	In motion	In motion	Stationery
	Light skin tissue distance	In contact			4.5	4.5	4.5	4.5	In contact
**Outcomes**	Method of conception	IVF			IVF	Natural (spontaneous)			
	Fertilised embryos	4	4	N/A	1	7	N/A	N/A	N/A
	Day 3 embryo	N/A	N/A	N/A	1	N/A	N/A	N/A	N/A
	Day 5 embryo	3	1	N/A	N/A	6	N/A	N/A	N/A
	PGT-A	2× Euploid	1× Euploid	N/A	N/A	6× Aneuploid	N/A	N/A	N/A
	Live birth	N/A	N/A	Yes	Yes	N/A	No	N/A	Yes

## Data Availability

All the data are provided in the text.
